# Artificial Intelligence Models in the Diagnosis of Adult-Onset Dementia Disorders: A Review

**DOI:** 10.3390/bioengineering9080370

**Published:** 2022-08-05

**Authors:** Gopi Battineni, Nalini Chintalapudi, Mohammad Amran Hossain, Giuseppe Losco, Ciro Ruocco, Getu Gamo Sagaro, Enea Traini, Giulio Nittari, Francesco Amenta

**Affiliations:** 1 Clinical Research Centre, School of Medicinal and Health Products Sciences, University of Camerino, 62032 Camerino, Italy; 2 School of Architecture and Design, University of Camerino, 63100 Ascoli Piceno, Italy

**Keywords:** adult-onset dementia, Alzheimer’s disease, magnetic resonance imaging, artificial intelligence, machine learning, neural networks

## Abstract

*Background:* The progressive aging of populations, primarily in the industrialized western world, is accompanied by the increased incidence of several non-transmittable diseases, including neurodegenerative diseases and adult-onset dementia disorders. To stimulate adequate interventions, including treatment and preventive measures, an early, accurate diagnosis is necessary. Conventional magnetic resonance imaging (MRI) represents a technique quite common for the diagnosis of neurological disorders. Increasing evidence indicates that the association of artificial intelligence (AI) approaches with MRI is particularly useful for improving the diagnostic accuracy of different dementia types. *Objectives:* In this work, we have systematically reviewed the characteristics of AI algorithms in the early detection of adult-onset dementia disorders, and also discussed its performance metrics. *Methods:* A document search was conducted with three databases, namely PubMed (Medline), Web of Science, and Scopus. The search was limited to the articles published after 2006 and in English only. The screening of the articles was performed using quality criteria based on the Newcastle–Ottawa Scale (NOS) rating. Only papers with an NOS score ≥ 7 were considered for further review. *Results:* The document search produced a count of 1876 articles and, because of duplication, 1195 papers were not considered. Multiple screenings were performed to assess quality criteria, which yielded 29 studies. All the selected articles were further grouped based on different attributes, including study type, type of AI model used in the identification of dementia, performance metrics, and data type. *Conclusions:* The most common adult-onset dementia disorders occurring were Alzheimer’s disease and vascular dementia. AI techniques associated with MRI resulted in increased diagnostic accuracy ranging from 73.3% to 99%. These findings suggest that AI should be associated with conventional MRI techniques to obtain a precise and early diagnosis of dementia disorders occurring in old age.

## 1. Introduction

Adult-onset cognitive disorders (AOCD) are characterized by a clinically significant, acquired impairment of cognitive functions [1,2]. Around 50 million people were affected by AOCD (dementia) worldwide in 2018, with a cost of approximately one trillion dollars for their care every year [3]. There is an impairment in daily functioning caused by multiple cognitive deficits. The main symptoms of AOCD are dementia, delirium, and mild cognitive impairment (MCI). A person with dementia has severe impairments in memory, language, problem solving, and other thinking abilities [4]. In most cases, delirium is defined as a state of acute disturbance of consciousness accompanied by a change in cognition during the day [5,6], whereas MCI is characterized by loss of memory and other cognitive abilities in individuals [7].

The impairment of neurocognitive function is associated with several neurological conditions, including Alzheimer’s disease (AD), frontotemporal dementia, Lewy body disease, Parkinson’s disease (PD), Huntington’s disease, Prion disease, traumatic brain injury, and others [8,9,10,11]. A pathophysiological correlation has been demonstrated between the progression of AD and nerve cell loss, neuro-fibrillary tangles, and senile plaques [12,13,14]. However, amyloid levels do not correlate directly with the progression of AD, affecting primarily the hippocampal, entorhinal cortex, neocortex, and other brain regions [12]. Neurofibrillary degeneration has been observed hierarchically among brain regions, and a pattern of progression of lesions is generally accepted [15]. 

Neurocognitive tests, brain imaging, and cerebrospinal fluid (CSF) tests are currently used to diagnose AD [16]. By improving diagnostics, biomarkers can facilitate early AD detection and treatment [17]. Studies have demonstrated the importance of early diagnostics, pharmacological interventions, lifestyle changes, and decreasing cardiovascular risk factors in suppressing the progression of the disease [18,19,20]. Therefore, it is imperative to diagnose clinical conditions that can potentially progress into dementia as early as possible [21,22]. 

In this 21st century, artificial intelligence (AI) composed of both machine learning (ML) and deep learning (DL) is rapidly revolutionizing the field of medicine [23]. ML involves an AI algorithm that selects the most suitable model based on a set of alternatives. For complex applications, ML algorithms have several advantages, including nonlinearity, fault tolerance, and real-time operation. Although the ML models incorporate information not ordinarily available to clinicians, such as advanced neuroimaging, genetic testing, and cerebrospinal fluid biomarkers, they can be applied to specialist and research settings [24].

Recent studies demonstrated the effectiveness of ML algorithms in neuroimaging and cognitive testing for the early detection of neurodegenerative diseases such as AD [25,26]. Patients with dementia will benefit from high-quality care when these diverse and strategic resources are utilized effectively. Therefore, ML is a crucial component in achieving this goal, and there is evidence that ML knowledge from clinical data can be used to plan care for people at risk of different dementia forms [27,28,29,30,31]. Review articles on the use of AI in the brain sciences analyze the opportunities and challenges associated with its implementation [32,33]. Neurogenerative disorders are poorly understood due to a lack of systematic analysis of AI technologies. 

This systematic review examines the involvement of AI applications in AOCDs. In this study, all performance metrics of the AI model for the early diagnosis of neurogenerative disorders such as dementia are presented. It provides a comprehensive overview of the state-of-the-art for machine learning about health informatics in dementia care. As we deal with big health data, we compile and review existing scientific methodologies. It has been demonstrated that ML can contribute to the analysis of neuroimaging data in dementia care. However, a relatively small effort has been made to apply advanced ML approaches to integrated heterogeneous data, which demonstrates the future potential and directions in dementia informatics.

## 2. Methods

### 2.1. Document Search

**Figure 1:**
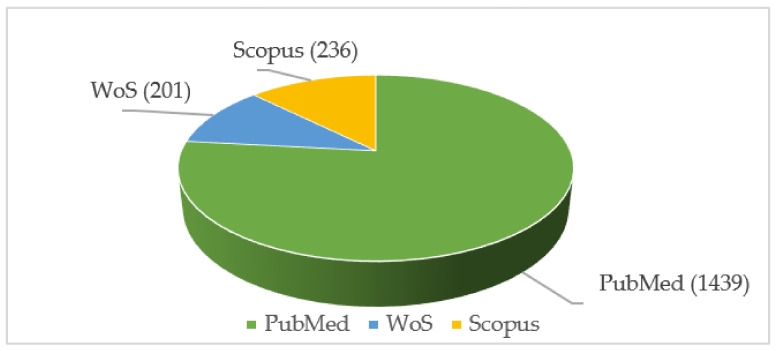
Document distribution of each database.

The review was conducted based on the guidelines of the Preferred Reporting Items for Systematic Reviews and Meta-Analyses (PRISMA) 2020. The document search was performed based on available literature from the databases PubMed, Web of Science (WoS), and Scopus. The document search was performed between the years 2006 and 2022. Articles before 2006 were excluded because of the limited literature on the topic of AI techniques in the diagnosis of neurogenerative diseases. Search keywords used were “artificial intelligence, “machine learning, “deep learning, “dementia”, “Alzheimer”s disease”, and “MRI”. The search queries were carefully framed using Medical Subject Headings (MeSH) for different databases, which are further listed in Table 1. The document distribution of each database can be found in Figure 1. 

### 2.2. Inclusion and Exclusion Criteria

We included all articles focused on AI use in dementia diagnosis or early-stage identification. The articles handling the data of patients with different types of dementia and those in the English language met the basic requirements of the inclusion criteria. The adoption of AI-related ML and DL model outcomes with 2 × 2 confusion matrix outcomes was considered. Papers published before 2006 and works not reporting the training and testing data split or not providing information on validation approaches were excluded. Papers published in languages other than English and dealing with animals were not considered either. Conference papers or proceedings with insufficient data on patients’ information, lack of information on the used model type, and validation approaches were excluded.

### 2.3. Quality Assessment

Once the literature search was carried out, the four authors independently assessed each article in two phases. In the first phase, similar or duplicate documents extracted from the three databases were eliminated by reading the abstracts. This analysis was conducted with the conventional approach of reading the article title and abstract. The inclusion and exclusion criteria of the filters were applied, and the evolution of the quality of each selected element was carried out based on the Newcastle–Ottawa scale (NOS), which varied from 0 to 9 [34]. The NOS defines each study in three ways: Poor (0–4), Moderate (5–6), and Good (7–9). These scores are based on some filters, such as study selection, comparability, and outcome. Various quality parameters, such as demonstration, coherence, risk factors, and others, are considered. The quality scores of selected articles depend on these parameters. These scores were recorded in an Excel sheet to calculate whether the selected study was suitable for final consideration or not.

## 3. Results

### 3.1. Search Outcomes

**Figure 2:**
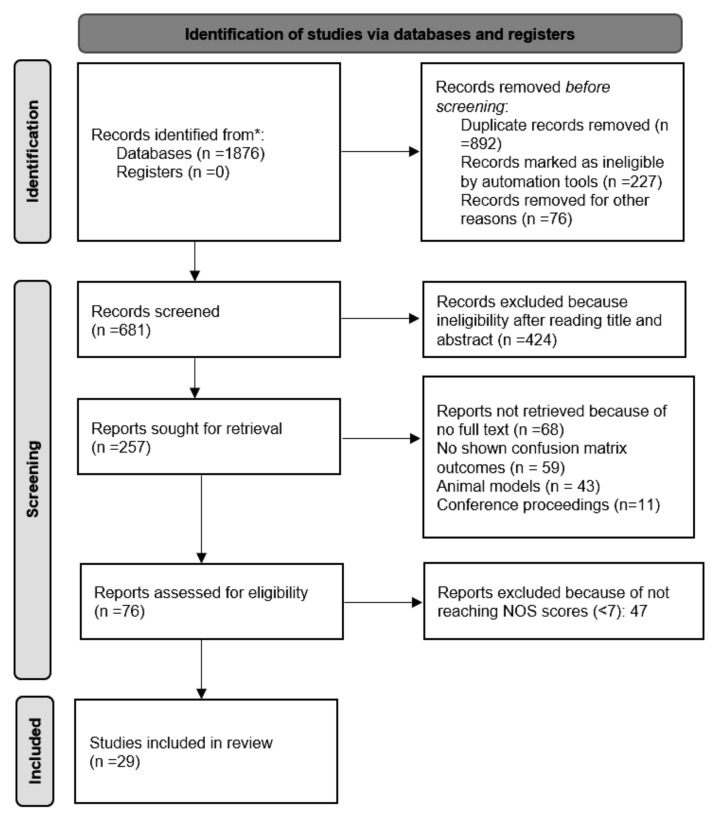
PRISMA 2020 flow chart for new systematic reviews with databases and registry search (*records extracted from only mentioned databases).

With a literature search, 1876 documents were identified in the period mentioned. Overall, 1195 documents were excluded due to duplication, ineligibility, and other reasons. This resulted in 681 documents being screened. Based on the title and abstract, 424 papers were excluded from further analysis as they were not consistent with the study objectives. At the end of the preliminary assessment, 257 works were considered for further review. For quality assessment, 76 documents were selected after applying inclusion and exclusion criteria. To perform multiple screenings, authors were given the selected documents and asked to note down quality scores anonymously for each work. In the absence of a high-quality score, items outside the review objectives were not further analyzed. We included 29 studies and summarized their findings in tabular form (Figure 2).

In terms of AI classifiers, 28.6% of the reported number of studies developed models using support vector machines (SVMs), and the models achieved accuracy ranging between 77.17% and 95.0%. In addition, two studies used Random Forest (RF) whereas the remaining eight studies used multiple AI classifiers [34,35]. In this review, we have found that AI models were used in five studies to diagnose AD, and six studies to diagnose other sorts of dementia. 

In most of the studies, AD detection is considered the highest priority. All these works are associated with neuroimage data such as MRI data with dual modes (demographic or image), positron emission tomography (PET), and other cognitive datasets. Studies with deep learning neural networks produced a maximum accuracy of 98.3% [35,36,37]. As shown in [38], the authors used neural network modeling to verify performance, and their results showed that DenseNet-121 generated accuracy of 90.22%, which is higher than Inception-V1, V2, and Residual Networks [39]. A simple classification model based on a decision tree with hyperparameter tuning produced 99% accuracy [40].

Two studies developed an AI model for diagnosing Parkinson’s disease. In one of these works, a CNN was trained and validated to detect PD from whole slide images (WSI). Model results show high accuracy, sensitivity, and specificity of 99%. Another paper developed an ML model for predicting Parkinson’s disease using the MRI method [41]. The model achieved 88% accuracy. The use of AI to diagnose and determine the prognosis of dementia was explored in three studies [42,43,44]. 

### 3.2. Study Characteristics

The main characteristics of the selected papers (investigated country, study type, dementia category, AI models and validation approaches, and performance metrics such as accuracy, sensitivity, and specificity) are summarized in Table 2. Among 29 selected works, a major part (22) of the studies are retrospective types, and the remaining seven are prospective cohort studies. Moreover, 17 works combine the involvement of MRI data coupling with AI modeling as a means of facilitating dementia and AD diagnosis [45,46,47,48,49,50,51]. Furthermore, electroencephalogram (EEG) sensors and clinical data can predict the risk of other dementia types, such as MCI, PD, and frontotemporal [52,53,54,55,56,57,58]. On other hand, it has been observed that nine studies appeared from the USA, which was followed by the UK (3), India (3), and Canada (2).

Various AI algorithms are used to assist in identifying different forms of dementia. Results mention that the common cause of neurocognitive disorders is AD, whose main features are progressive memory loss and multidomain cognitive decline. AD represents 60% of all neurocognitive disorders [59]. AOCDs are a major cause of disability in the general population. Current data and prospects make dementia treatment a pivotal topic in the planning of national health systems, recognizing it as a major challenge for proposing sustainable choices for health and social assistance [60].

In terms of AI classifiers, 28.6% of studies applied SVM models and achieved accuracy in between 77.17% and 95.0% [49,50,56,58]. In addition, two studies used RF algorithms [45,51], whereas the remaining eight used multiple AI classifiers [46,47,48,52,53,54,55,57]. The present review found that five studies used AI models in AD diagnosis [45,46,47,52,56], and six studies to diagnose other dementia types [48,49,50,53,57]. 

Two studies developed an AI model for PD diagnosis [41,61]. A Boutet et al. developed an ML model for PD prediction using the MRI method [41], and M Signaevsky et al. trained and validated a CNN to detect PD from whole slide images (WSI) [61]. Results show high accuracy, sensitivity, and specificity. CI and MCI were classified with 81% and 96.6% of accuracy with recurrent neural networks (RNN) and artificial neural networks (ANN), respectively [43,62]. A study using multi-layer perceptrons (MLP) with cognitive data showed that 92.98% of AD cases were accurately diagnosed [63]. The mini-mental state examination (MMSE) and clinical dementia ratio (CDR) tests were also used to further classify AD stages with ResNet and DenseNet, which resulted in 99% accuracy [64,65]. 

## 4. Discussion

Our study reviewed the research literature on the application of AI models in the early detection of dementia in adults. A review of outcome data has shown that AI or ML models can greatly influence any subspecialty within AOCD at every treatment stage. To predict dementia types in advance, ANN, MRI data, and labeling segments have been most frequently used.

### 4.1. AI for Diagnostic Purposes

Currently, the treatment of AOCDs is limited to symptomatic therapies available, and drugs used in the treatment of dementias have very limited therapeutic value. For this reason, advanced computing techniques such as AI, ML, and deep learning have been directed toward the search for non-pharmacological approaches and support for caregivers [18]. It is now widely accepted that the phase of overt dementia in AD is preceded by a long preclinical phase, sometimes lasting several decades, that evolves through a continuum, from the initial preclinical stages to MCI up to the overt clinical stage of dementia [66,67]. People with advanced dementia have similar outcomes with psychosocial interventions as with pharmacological interventions. It has been demonstrated that cognitive stimulation improves cognition as well as the self-reported quality of life (QOL) and wellbeing. Computer-assisted exercise has been linked to better QOL for people with disabilities; however, not much research has been conducted. A pilot study examined whether computer-assisted exergaming interventions, utilizing exergaming technology (Able-X), could improve QOL, including cognitive and physical functioning, in 10 dementia patients, in addition to existing therapies and activities [68]. The role of AI algorithms in effectively detecting the different AOCD types was explained further.

A. MCI detection

MCI is considered a transitional phase between normal aging and dementia [7]. When compared with nondepressed patients with MCI, individuals with MCI and depression perform less well on immediate and delayed memory tasks. MCI patients who experience sub-syndromic symptoms of depression have been found to have poorer function and quality of life, as well as a higher risk of dementia progression. Therefore, those who are cognitively impaired must undergo appropriate screening strategies for depression and depressive symptoms. This will enable clinicians to identify the causes of cognitive, functional, and behavioral impairments. It is thought that, in this phase, it is possible to intervene and slow the progression versus overt dementia during this stage. In this systematic review, four studies employed ML models to detect MCI [42,43,58]. An SVM model was the most incorporated algorithm in the detection of MCI and produced accuracy ranging from 73% to 91% [56,58]. Advanced ML models such as ANN can have the ability to detect MCI with 96.66% accuracy [43].

B. AD diagnosis

AD is a brain neurodegenerative disorder occurring mainly in diseases commonly affecting elderly people, although it is not a normal part of aging. As AD progresses, memory loss, personality changes, and changes in brain function gradually worsen. AD is the most common adult-onset dementia. In this review, we found that 16 studies out of 29 (55%) used AI models to diagnose AD. According to these studies, AI models performed well in detecting AD, with an accuracy range of 73.33–99%, a sensitivity range of 70.8–90.10%, and a specificity range of 70–90%. A total of 11 studies (70%) utilized AI in conjunction with magnetic resonance imaging (MRI) to diagnose AD. Two studies analyzed clinical data, one along with MRI. One study used positron emission tomography (PET) and MRI. The remaining research used EEG and cognitive data to diagnose AD with AI models.

C. Frontotemporal (FTD) and Lewy bodies (LBD) dementia

To target interventions and treatments for frontotemporal dementia (FTD), an accurate differential diagnosis is vital [69]. There are studies suggesting that deep learning techniques can be used to solve the differential diagnosis problem for FTD, AD, and normal controls (NCs), but their performance is still unknown. A third issue is that existing DL-assisted diagnostic studies are still reliant on expert-level preprocessing based on hypotheses. Some ML tools help to distinguish the AD and FTD symptoms with genetic algorithms [70]. It has been demonstrated that a data-centric perspective helps to understand AD and FTD disorders by allowing the results to be interpreted. 

While LBD is a dementia-type syndrome with many clinical similarities, it can be difficult to diagnose clinically, especially in the advanced stages. To identify these disorders with a high prognosis, researchers proposed an ML algorithm based solely on non-invasive and easily collectable predictors [71]. The ImageNet dataset and ADNI database were used to reduce model complexity based on two-stage transfer learning technology [72,73]. Using the medical experience as a concatenation layer in the deep learning model, the AI model can automatically extract features corresponding to regulation and domain knowledge. Using this approach, the deep learning model gains better training efficiency and identifies more significant features in differentiating AD and LBD.

D. PD diagnosis

PD is a neurological disease characterized by shaking, stiffness, and difficulties in walking, balance, and coordination. Symptoms usually develop gradually. People may have trouble walking and talking as the disease progresses. In addition, they may have psychological changes, sleeping problems, depression, and memory issues. In this systematic review, five studies associated PD detection with AI algorithms with MRI, clinical data, and WSI. They reported an accuracy range of 74–99%, a sensitivity range of 68.4–99%, and a specificity range of 70–99% for their developed AI models in PD diagnosis. 

### 4.2. Model Assessment

Various AI algorithms are used to assist in identifying different forms of dementia in this section. There were two groups of AI algorithms, including ML and DL, reviewed in this work. Eighteen studies employed traditional ML classifiers, among which four utilized SVM, with accuracy ranging from 77.17% to 95.0% [49,50,56,58]. In addition, two studies applied RF [45,51], and one study employed Random Under-Sampling RF (RUSRF) [39], with an accuracy range of 73.3% to 94.4%. ML models were employed by G. Lee et al. [62], without mentioning any particular algorithm’s name, and showed 88% accuracy. Using multilayer perceptron (MLP) modeling, AD classification with 92.98% of accuracy was achieved [63]. In [40], the authors developed a model using the decision tree classifier with hyperparameter tuning (DTC-HPT) and observed high accuracy of 99% for identifying AD. On the other hand, the remaining eight studies applied multiple ML classifiers [46,47,48], and they performed extremely well, with an accuracy range of 68% to 99.1% [52,53,54,55,57].

DL classifiers were used in nine (31%) of the 29 studies reviewed. Four of the selected studies employed conventional neural networks (CNNs) [35,36,37,61], reaching the highest accuracy of 99% and the lowest accuracy of 84%. ANN [43] and RNN [62] were used in two studies, with results of 96.66% and 81%, respectively. Three of the remaining studies compared multiple DL models [38,44,65], with accuracy ranging from 59.8% to 98.86%. Two studies were associated with both ML and DL classifiers [38,64]. A model using SVM and a second using a combination of MobileNet and Block 11 addition and SVM were noted [42]. In terms of accuracy, the combined model had the highest accuracy of 88.7%, while the SVM model had the lowest accuracy of 73.3%. A gradient-boosting model (GBM) as well as a Residual Neural Network (ResNet-50) have been designed by authors [64] and showed 91.3% and 98.99% accuracy.

### 4.3. Research Implications

Dementia is not a specific disease—it is a group of symptoms severely affecting memory loss, thinking, decision making, and social abilities so as to interfere with daily life. Several diseases can cause dementia. The prevalence of dementia increases with age, but it is not a normal part of aging. Symptoms vary according to the type of dementia. In this analysis, there were ten studies (33%) that developed different types of AI models to detect dementia by analyzing MRI data (40%), EEG facial expressions, NPT, and clinical and voice records. The performance of the AI model was evaluated in terms of accuracy (range of 74–99.1%), sensitivity (range of 66.3–99%), and specificity (58–99%). It is now widely accepted that the phase of overt dementia in AD is preceded by a long preclinical phase, sometimes lasting several decades, that evolves through a continuum, from the initial preclinical stages to MCI up to the overt clinical stage of dementia [66,67].

Current AI algorithms are recognized with measurable consistencies in large datasets and are routinely utilized across a scope of different domains, including disease diagnosis, but these models lack the power and generalizability related to human learning. If AI procedures could empower computers to self-learn from fewer examples, the experimental outcomes could have comprehensive logical and cultural effects. With increased memory and increased processing power, large models can provide more sophisticated outcomes and more adaptable learning. It is becoming increasingly clear that substantially more prominent figuring assets will not suffice to produce calculations suitable for learning from a few prototypes and summing up past preparation sets. Shortly, we may be able to distinguish dementia from normal aging by using movement tests and smart environments. Future directions to improve dementia detection in its earliest stages could include AI-based smart environments and multimodal examinations. 

### 4.4. Limitations

The current work has a few important limitations that need to be addressed. First, the database search did not capture all the related papers; thus, it could not obtain all the eligible articles as a whole. The search terms mentioned in this work could be insufficient to identify the whole literature on AI combined with dementia. We highlighted the detection of adult-onset dementia disorders and ML and DL algorithms associated with it. This led to missing studies on working life dementia. On the other hand, in this review, we adopted only three major databases. This limited the coverage of other journals that are in line with the research topic.

## 5. Conclusions

Medicine is undergoing a revolution because of AI and ML, which help in the diagnosis of any disease, making it easier in recent years. With a more precise diagnosis, this technology could transform healthcare. A computerized system helps doctors to diagnose patients more accurately, predict what patients’ future health will look like, and recommends better treatments. In this review, we have investigated current approaches of AI in the diagnosis and early prediction of adult-onset dementia disorders. In the past, dementia diagnosis was performed solely based on correlations between symptoms and the most likely cause. The newly developed methods with AI overcome several conventional limitations by utilizing causal reasoning in their machine learning. As a result of AI, dementia screening can now be automated to an even higher degree. This is particularly appealing to epidemiology studies and public health organizations that aim to target early risk reduction interventions. In contrast to clinicians’ judgment alone, AI can analyze and respond quickly to large population screenings. 

## Figures and Tables

**Table 1:** Search queries for three adopted databases.

Database	Query
PubMed	English AND (“Artificial Intelligence” [Title/Abstract/MeSH] OR “Machine Learning”[Title/Abstract/MeSH]) OR “Deep learning” AND (“diagnosis”[Title/Abstract] OR “detection”[Title/Abstract] OR “identification”[Title/Abstract] OR “recognition”[Title/Abstract]) OR “interpretation”[Title/Abstract]) AND (“dementia”[All Fields] AND “MRI”[All Fields]) AND “PET” [All Fields]) AND “image data”[All Fields]) NOT “classification” [Title/Abstract/MeSH] NOT “ranking”[Title/Abstract/MeSH] NOT “grouping”[Title/Abstract/MeSH] NOT Review[ptyp] NOT books and Documents [ptyp] NOT conference [ptyp]
WoS	(“AI” AND “Artificial Intelligence” AND “Machine Learning” AND “Deep Learning”) AND (“Diagnosis” OR “Identification” OR “recognition”) AND (“dementia” OR “Alzheimer’s disease” OR “MRI” OR “PET” OR “medical imaging” OR “neuro”) NOT “segmentation” NOT “functional” NOT “connectivity”) AND LANGUAGE: (English) AND DOCUMENT TYPES: (Review OR Proceedings Paper)
Scopus	TITLE-ABS-KEY (“Artificial Intelligence” AND “Machine Learning” AND “Deep Learning”) AND (“Diagnosis” OR “Identification” OR “recognition” OR “interpretation) AND (“neurological diseases” OR “neurogenerative disorders” OR “dementia” OR “MRI” OR “PET”) AND LIMIT-TO (LANGUAGE, “English”) AND (LIMIT-TO (EXACT KEYWORD, “dementia”)

**Table 2:** Characteristics of papers included in the review.

N	Country	Study Cohort	Dementia Category	AI Model	AI Modality	Validation Methods	Accuracy	Sensitivity	Specificity	Ref.
1	Canada	Prospective	AD	RUSRF	PET, MRI	Independent test set	84%	70.8%	86.5%	[39]
2	UK, China	Retrospective	MCI, Dementia	MobileNet, SVM	Facial expressions	5-fold cross-validation	73.3%	N/A	N/A	[42]
3	India	Retrospective	AD	DNN, Inception-V1, V2, V3, Residual Networks, DenseNet	MRI	Independent test set	90.22%	N/A	N/A	[38]
4	India	Retrospective	AD	CNN	MRI	Independent test set	98.3%	97%	N/A	[35]
5	India	Retrospective	AD	DTC-HPT	MRI	Independent test set	99%	99.10%	N/A	[40]
6	Egypt	Retrospective	AD	CNN	MRI	10-fold cross-validation	97%	95%	N/A	[36]
7	USA	Retrospective	AD	ResNet-50, GBM	MRI	10-fold cross-validation	99%	N/A	N/A	[64]
8	USA	Retrospective	AD	MLP	Cognitive data	Independent test set	92.98%	93.75%	92.68%	[63]
9	Canada	Retrospective	AD	CNN	MRI	5-fold cross-validation	84%	N/A	N/A	[37]
10	South Korea	Retrospective	MCI, Dementia	ANN	NPT data	10-fold cross-validation	96.66%	96%	96.8%	[43]
11	USA	Prospective	Dementia	LSTM, CNN	Voice Data	5-fold cross-validation	74%	66.3%	84.7%	[44]
12	USA	Prospective	PD	CNN	WSI	Cross-validation	99%	99%	99%	[61]
13	USA	Prospective	AD	RNN	MRI	5-fold cross-validation	81%	84%	80%%	[62]
14	Lithuania	Retrospective	AD	ResNet18, DenseNet201	MRI	Cross-validation	98.86%	98.89%	N/A	[65]
15	Canada	Prospective	PD	ML model	MRI	Independent test set/ 5-fold cross-validation	88%	N/A	N/A	[41]
16	Spain	Retrospective	AD	RF	MRI	Cross-validation	94.4%	N/A	N/A	[45]
17	Greece	Retrospective	AD and Frontotemporal Dementia	DT, RF, ANN, SVM, Naïve Bayes, and KNN	EEG	10-fold and leave-one-patient-out cross-validation	80% (DT)–99.1% (RF)	94% (NB)–98.6% (RF)	58% (NB)–99% (RF)	[52]
18	Italy	Retrospective	AD	Gradient boosting, SVM, LR, RF, AdaBoosting, NB	MRI	Cross-validation	95.96% (NB)–97.58% (GB)	95%–96%	N/A	[46]
19	UK	Retrospective	Dementia	RF and XGBoost	Clinical data	5-fold cross-validation	85% (RF)–87% (XGB)	73% (RF)–76% (XGB)	99% (RF) and (XGB)	[53]
20	USA	Retrospective	PD	Classification tree, Gaussian Kernel, LDA, Ensemble, KNN, LR, Naive Bayes, SVM, RF	Clinical data	Leave-one-subject-out cross-validation	74.1% (SVM)–84.5% (KNN)	70.6% (SVM)–88.5% (KNN)	79.2% (SVM)–84.6% (LR)	[54]
21	USA	Retrospective	AD	KNN, SVM, DT, RF, DL	MRI, SNP, clinical data	Internal cross-validation and an external test set	68% (KNN)–89%(DL)	N/A	N/A	[47]
22	Italy	Retrospective	PD	SVM, KNN, LDA, LR	Clinical data	10-fold cross-validation	90.1% (LDA)–91.8% (SVM)	68.4% (SVM)–87.5% (SVM optimized cost)	N/A	[55]
23	UK	Retrospective	Dementia	NB, LD, SVM, and KNN	MRI	10-fold cross-validation	77% (NB)–93% (C-SVM)	72.5% (CNN)–99% (KNN)	67% (KNN)–95% (SVM)	[48]
24	Netherlands	Retrospective	Dementia	Linear SVM	MRI, PET	LOO cross-validation and four-fold cross-validation	89% (voxel)–90% (Region)	83% (Region)–85% (voxel)	79% (voxel)–90% (Region)	[49]
25	Finland	Prospective	Dementia	SVM	MRI/CT, clinical data	5-fold cross-validation	95%	93%	99%	[50]
26	Japan	Retrospective	Dementia	XGBoost, RF, LR	Clinical data	-	86.3% (XGBoost)–89.3% (LR)	85.7% (XGBoost)–96.4% (LR)	80.0% (RF)–89.3% (LR)	[57]
27	USA	Retrospective	MCI and AD	SVM	Clinical data	5-fold cross-validation	91%	N/A	N/A	[56]
28	USA	Prospective	MCI	SVM	Clinical data	5-fold cross-validation	77.17%	81.97%	67.74%	[58]
29	Korea	Retrospective	AD and PD	RF	MRI	5-fold cross-validation	73.3%	78.0%	70.0%	[51]

## Data Availability

Not applicable.

## Abbreviations

AD: Alzheimer’s disease; PD: Parkinson’s disease; MCI: Mild cognitive impairment; MRI: Magnetic resonance imaging; PET: Positron emission tomography; CT: Computed tomography; ML: Machine learning; AI: Artificial intelligence; NB: Naïve Bayes; RF: Random Forest; ANN: Artificial neural network; RNN: Recurrent neural network; KNN: K-Nearest Neighborhood; SVM: Support vector machine; DT: Decision tree; NN: Neural network; LR: Logistic regression; RUSRF: Random Under-Sampling Random Forest; CNN: Conventional neural network; DNN: Deep neural network; DTC-HPT: Decision tree classifier with hyperparameter tuning; ResNet: Residual Network; GBM: Gradient boosting classifier; MLP: Multilayer perception; LSTM: Long Short-Term Memory; XGBOOST: eXtreme Gradient Boosting; LDA: Linear discriminant analysis.

## References

[1] Harrison R.A., Kesler S.R., Johnson J.M., Penas-Prado M., Sullaway C.M., Wefel J.S. (2019). Neurocognitive dysfunction in adult cerebellar medulloblastoma. Psycho-Oncology.

[2] Chang K.J., Zhao Z., Shen H.R., Bing Q., Li N., Guo X., Hu J. (2021). Adolescent/adult-onset homocysteine remethylation disorders characterized by gait disturbance with/without psychiatric symptoms and cognitive decline: A series of seven cases. Neurol. Sci..

[3] Dubois B., Villain N., Frisoni G.B., Rabinovici G.D., Sabbagh M., Cappa S., Bejanin A., Bombois S., Epelbaum S., Teichmann M. (2021). Clinical diagnosis of Alzheimer’s disease: Recommendations of the International Working Group. Lancet. Neurol..

[4] Spiegel D., Lewis-Ferńandez R., Lanius R., Vermetten E., Simeon D., Friedman M. (2013). Dissociative disorders in DSM-5. Annu. Rev. Clin. Psychol..

[5] Gnerre P., La Regina M., Bozzano C., Pomero F., Re R., Meschi M., Montemurro D., Marchetti A., Di Lillo M., Tirotta D. (2016). Delirium: The invisible syndrome. Ital. J. Med..

[6] Bhat R., Rockwood K. (2007). Delirium as a disorder of consciousness. J. Neurol. Neurosurg. Psychiatry.

[7] Smith G.E., Bondi M.W. (2013). Mild Cognitive Impairment and Dementia: Definitions, Diagnosis, and Treatment.

[8] Vahia V.N. (2013). Diagnostic and statistical manual of mental disorders 5: A quick glance. Indian J. Psychiatry..

[9] Dening T., Sandilyan M.B. (2015). Dementia: Definitions and types. Nurs. Stand..

[10] Aarsland D. (2020). Epidemiology and Pathophysiology of Dementia-Related Psychosis. J. Clin. Psychiatry.

[11] Ferencz B., Gerritsen L. (2015). Genetics and Underlying Pathology of Dementia. Neuropsychol. Rev..

[12] Ingelsson M., Fukumoto H., Newell K.L., Growdon J.H., Hedley-Whyte E.T., Frosch M.P., Albert M.S., Hyman B.T., Irizarry M.C. (2004). Early Abeta accumulation and progressive synaptic loss, gliosis, and tangle formation in AD brain. Neurology.

[13] Serrano-Pozo A., Mielke M.L., Gómez-Isla T., Betensky R.A., Growdon J.H., Frosch M.P., Hyman B.T. (2011). Reactive glia not only associates with plaques but also parallels tangles in Alzheimer’s disease. Am. J. Pathol..

[14] Serrano-Pozo A., Frosch M.P., Masliah E., Hyman B.T. (2011). Neuropathological Alterations in Alzheimer Disease. Cold Spring Harb. Perspect. Med..

[15] Wisniewski H.M., Silverman W. (1997). Diagnostic criteria for the neuropathological assessment of Alzheimer’s disease: Current status and major issues. Neurobiol. Aging.

[16] McKhann G.M., Knopman D.S., Chertkow H., Hyman B.T., Jack C.R., Kawas C.H., Klunk W.E., Koroshetz W.J., Manly J.J., Mayeux R. (2011). The diagnosis of dementia due to Alzheimer’s disease: Recommendations from the National Institute on Aging-Alzheimer’s Association workgroups on diagnostic guidelines for Alzheimer’s disease. Alzheimer’s Dement..

[17] Jack C.R., Bennett D.A., Blennow K., Carrillo M.C., Dunn B., Haeberlein S.B., Holtzman D.M., Jagust W., Jessen F., Karlawish J. (2018). NIA-AA Research Framework: Toward a biological definition of Alzheimer’s disease. Alzheimer’s Dement..

[18] Livingston G., Sommerlad A., Orgeta V., Costafreda S.G., Huntley J., Ames D., Ballard C., Banerjee S., Burns A., Cohen-Mansfield J. (2017). Dementia prevention, intervention, and care. Lancet.

[19] Maki Y., Yamaguchi H. (2014). Early detection of dementia in the community under a community-based integrated care system. Geriatr. Gerontol. Int..

[20] Arevalo-Rodriguez I., Smailagic N., Roqué-Figuls M., Ciapponi A., Sanchez-Perez E., Giannakou A., Pedraza O.L., Bonfill Cosp X., Cullum S. (2021). Mini-Mental State Examination (MMSE) for the early detection of dementia in people with mild cognitive impairment (MCI). Cochrane Database Syst. Rev..

[21] Battineni G., Hossain M.A., Chintalapudi N., Traini E., Dhulipalla V.R., Ramasamy M., Amenta F. (2021). Improved Alzheimer’s Disease Detection by MRI Using Multimodal Machine Learning Algorithms. Diagnostics.

[22] Carotenuto A., Traini E., Fasanaro A.M., Battineni G., Amenta F. (2021). Tele-Neuropsychological Assessment of Alzheimer’s Disease. J. Pers. Med..

[23] Woźniacka A., Patrzyk S., Mikołajczyk M. (2021). Artificial intelligence in medicine and dermatology. Postep. Dermatol. Alergol..

[24] James C., Ranson J.M., Everson R., Llewellyn D.J. (2021). Performance of Machine Learning Algorithms for Predicting Progression to Dementia in Memory Clinic Patients. JAMA Netw. Open.

[25] Herraiz Á.H., Martínez-Rodrigo A., Bertomeu-González V., Quesada A., Rieta J.J., Alcaraz R. (2020). A Deep Learning Approach for Featureless Robust Quality Assessment of Intermittent Atrial Fibrillation Recordings from Portable and Wearable Devices. Entropy.

[26] Gaubert S., Houot M., Raimondo F., Ansart M., Corsi M.C., Naccache L., Sitt J.D., Habert M.O., Dubois B., De Vico Fallani F. (2021). A machine learning approach to screen for preclinical Alzheimer’s disease. Neurobiol. Aging.

[27] Tsang G., Xie X., Zhou S.M. (2020). Harnessing the Power of Machine Learning in Dementia Informatics Research: Issues, Opportunities, and Challenges. IEEE Rev. Biomed. Eng..

[28] Kumar S., Oh I., Schindler S., Lai A.M., Payne P.R.O., Gupta A. (2021). Machine learning for modeling the progression of Alzheimer disease dementia using clinical data: A systematic literature review. JAMIA Open.

[29] Agarwal D., Marques G., De la Torre-Díez I., Franco Martin M.A., García Zapiraín B., Martín Rodríguez F. (2021). Transfer Learning for Alzheimer’s Disease through Neuroimaging Biomarkers: A Systematic Review. Sensors.

[30] Merkin A., Krishnamurthi R., Medvedev O.N. (2022). Machine learning, artificial intelligence and the prediction of dementia. Curr. Opin. Psychiatry.

[31] Landolfi A., Ricciardi C., Donisi L., Cesarelli G., Troisi J., Vitale C., Barone P., Amboni M. (2021). Machine Learning Approaches in Parkinson’s Disease. Curr. Med. Chem..

[32] Savage N. (2019). How AI and neuroscience drive each other forwards. Nature.

[33] Fan J., Fang L., Wu J., Guo Y., Dai Q. (2020). From brain science to artificial intelligence. Engineering.

[34] Stang A. (2010). Critical evaluation of the Newcastle-Ottawa scale for the assessment of the quality of nonrandomized studies in meta-analyses. Eur. J. Epidemiol..

[35] Goenka N., Tiwari S. (2022). AlzVNet: A volumetric convolutional neural network for multiclass classification of Alzheimer’s disease through multiple neuroimaging computational approaches. Biomed. Signal Process. Control.

[36] Helaly H.A., Badawy M., Haikal A.Y. (2021). Deep Learning Approach for Early Detection of Alzheimer’s Disease. Cognit. Comput..

[37] Pan D., Zeng A., Jia L., Huang Y., Frizzell T., Song X. (2020). Early Detection of Alzheimer’s Disease Using Magnetic Resonance Imaging: A Novel Approach Combining Convolutional Neural Networks and Ensemble Learning. Front. Neurosci..

[38] Hazarika R.A., Kandar D., Maji A.K. (2021). An experimental analysis of different Deep Learning based Models for Alzheimer’s Disease classification using Brain Magnetic Resonance Images. J. King Saud Univ.-Comput. Inf. Sci..

[39] Mathotaarachchi S., Pascoal T.A., Shin M., Benedet A.L., Kang M.S., Beaudry T., Fonov V.S., Gauthier S., Rosa-Neto P. (2017). Identifying incipient dementia individuals using machine learning and amyloid imaging. Neurobiol. Aging.

[40] Naganandhini S., Shanmugavadivu P. (2019). Effective Diagnosis of Alzheimer’s Disease using Modified Decision Tree Classifier. Procedia Comput. Sci..

[41] Boutet A., Madhavan R., Elias G.J.B., Joel S.E., Gramer R., Ranjan M., Paramanandam V., Xu D., Germann J., Loh A. (2021). Predicting optimal deep brain stimulation parameters for Parkinson’s disease using functional MRI and machine learning. Nat. Commun..

[42] Fei Z., Yang E., Yu L., Li X., Zhou H., Zhou W. (2022). A Novel deep neural network-based emotion analysis system for automatic detection of mild cognitive impairment in the elderly. Neurocomputing.

[43] Kang M.J., Kim S.Y., Na D.L., Kim B.C., Yang D.W., Kim E.J., Na H.R., Han H.J., Lee J.H., Kim J.H. (2019). Prediction of cognitive impairment via deep learning trained with multi-center neuropsychological test data. BMC Med. Inform. Decis. Mak..

[44] Xue C., Karjadi C., Paschalidis I.C., Au R., Kolachalama V.B. (2021). Detection of dementia on voice recordings using deep learning: A Framingham Heart Study. Alzheimer’s Res. Ther..

[45] El-Sappagh S., Alonso J.M., Islam S.M.R., Sultan A.M., Kwak K.S. (2021). A multilayer multimodal detection and prediction model based on explainable artificial intelligence for Alzheimer’s disease. Sci. Rep..

[46] Battineni G., Chintalapudi N., Amenta F., Traini E. (2020). A Comprehensive Machine-Learning Model Applied to Magnetic Resonance Imaging (MRI) to Predict Alzheimer’s Disease (AD) in Older Subjects. J. Clin. Med..

[47] Venugopalan J., Tong L., Hassanzadeh H.R., Wang M.D. (2021). Multimodal deep learning models for early detection of Alzheimer’s disease stage. Sci. Rep..

[48] Herzog N.J., Magoulas G.D. (2021). Brain asymmetry detection and machine learning classification for diagnosis of early dementia. Sensors.

[49] Bron E.E., Steketee R.M.E., Houston G.C., Oliver R.A., Achterberg H.C., Loog M., Van Swieten J.C., Hammers A., Niessen W.J., Smits M. (2014). Diagnostic classification of arterial spin labeling and structural MRI in presenile early stage dementia. Hum. Brain Mapp..

[50] Pekkala T., Hall A., Lötjönen J., Mattila J., Soininen H., Ngandu T., Laatikainen T., Kivipelto M., Solomon A. (2017). Development of a late-life dementia prediction index with supervised machine learning in the population-based CAIDE study. J. Alzheimer’s Dis..

[51] Byeon H. (2020). Application of machine learning technique to distinguish parkinson’s disease dementia and alzheimer’s dementia: Predictive power of parkinson’s disease-related non-motor symptoms and neuropsychological profile. J. Pers. Med..

[52] Miltiadous A., Tzimourta K.D., Giannakeas N., Tsipouras M.G., Afrantou T., Ioannidis P., Tzallas A.T. (2021). Alzheimer’s disease and frontotemporal dementia: A robust classification method of eeg signals and a comparison of validation methods. Diagnostics.

[53] Danso S.O., Zeng Z., Muniz-Terrera G., Ritchie C.W. (2021). Developing an Explainable Machine Learning-Based Personalised Dementia Risk Prediction Model: A Transfer Learning Approach With Ensemble Learning Algorithms. Front. Big Data.

[54] Juutinen M., Wang C., Zhu J., Haladjian J., Ruokolainen J., Puustinen J., Vehkaoja A. (2020). Parkinson’s disease detection from 20-step walking tests using inertial sensors of a smartphone: Machine learning approach based on an observational case-control study. PLoS ONE.

[55] Sabry F., Eltaras T., Labda W., Alzoubi K., Malluhi Q. (2022). Machine Learning for Healthcare Wearable Devices: The Big Picture. J. Healthc. Eng..

[56] Ghoraani B., Boettcher L.N., Hssayeni M.D., Rosenfeld A., Tolea M.I., Galvin J.E. (2021). Detection of Mild Cognitive Impairment and Alzheimer’s Disease using Dual-task Gait Assessments and Machine Learning Behnaz. Physiol. Behav..

[57] Shimoda A., Li Y., Hayashi H., Kondo N. (2021). Dementia risks identified by vocal features via telephone conversations: A novel machine learning prediction model. PLoS ONE.

[58] Boettcher L.N., Hssayeni M., Rosenfeld A., Tolea M.I., Galvin J.E., Ghoraani B. (2020). Dual-Task Gait Assessment and Machine Learning for Early- detection of Cognitive Decline. Physiol. Behav..

[59] Alzheimer’s Association (2016). 2016 Alzheimer’s disease facts and figures. Alzheimer’s Dement..

[60] WHO (2019). Risk Reduction of Cognitive Decline and Dementia: WHO Guidelines.

[61] Signaevsky M., Marami B., Prastawa M., Tabish N., Iida M.A., Zhang X.F., Sawyer M., Duran I., Koenigsberg D.G., Bryce C.H. (2022). Antemortem detection of Parkinson’s disease pathology in peripheral biopsies using artificial intelligence. Acta Neuropathol. Commun..

[62] Lee G., Nho K., Kang B., Sohn K.A., Kim D., Weiner M.W., Aisen P., Petersen R., Jack C.R., Jagust W. (2019). Predicting Alzheimer’s disease progression using multi-modal deep learning approach. Sci. Rep..

[63] Almubark I., Chang L.C., Shattuck K.F., Nguyen T., Turner R.S., Jiang X. (2020). A 5-min Cognitive Task With Deep Learning Accurately Detects Early Alzheimer’s Disease. Front. Aging Neurosci..

[64] Fulton L.V., Dolezel D., Harrop J., Yan Y., Fulton C.P. (2019). Classification of alzheimer’s disease with and without imagery using gradient boosted machines and resnet-50. Brain Sci..

[65] Odusami M., Maskeliūnas R., Damaševičius R. (2022). An Intelligent System for Early Recognition of Alzheimer’s Disease Using Neuroimaging. Sensors.

[66] Dubois B., Hampel H., Feldman H.H., Scheltens P., Aisen P., Andrieu S., Bakardjian H., Benali H., Bertram L., Blennow K. (2016). Preclinical Alzheimer’s disease: Definition, natural history, and diagnostic criteria. Alzheimer’s Dement..

[67] Scheltens P., Blennow K., Breteler M.M.B., De Strooper B., Frisoni G.B., Salloway S., Van der Flier W.M. (2016). Alzheimer’s disease. Lancet.

[68] Drury-Ruddlesden J., Health I. (2017). Rehabilitation in Advanced Dementia through Computer-Assisted Exergaming with Able-X: A Collective Case Study. Ph.D. Thesis.

[69] Hu J., Qing Z., Liu R., Zhang X., Lv P., Wang M., Wang Y., He K., Gao Y., Zhang B. (2021). Deep Learning-Based Classification and Voxel-Based Visualization of Frontotemporal Dementia and Alzheimer’s Disease. Front. Neurosci..

[70] García-Gutierrez F., Díaz-Álvarez J., Matias-Guiu J.A., Pytel V., Matías-Guiu J., Cabrera-Martín M.N., Ayala J.L. (2022). GA-MADRID: Design and validation of a machine learning tool for the diagnosis of Alzheimer’s disease and frontotemporal dementia using genetic algorithms. Med. Biol. Eng. Comput..

[71] Bougea A., Efthymiopoulou E., Spanou I., Zikos P. (2022). A Novel Machine Learning Algorithm Predicts Dementia With Lewy Bodies Versus Parkinson’s Disease Dementia Based on Clinical and Neuropsychological Scores. J. Geriatr. Psychiatry Neurol..

[72] Galvin J.E., Chrisphonte S., Cohen I., Greenfield K.K., Kleiman M.J., Moore C., Riccio M.L., Rosenfeld A., Shkolnik N., Walker M. (2021). Characterization of dementia with Lewy bodies (DLB) and mild cognitive impairment using the Lewy body dementia module (LBD-MOD). Alzheimer’s Dement..

[73] Ni Y.C., Tseng F.P., Pai M.C., Hsiao I.T., Lin K.J., Lin Z.K., Lin C.Y., Chiu P.Y., Hung G.U., Chang C.C. (2021). The Feasibility of Differentiating Lewy Body Dementia and Alzheimer’s Disease by Deep Learning Using ECD SPECT Images. Diagnostics.

